# Antifungal Activity of Essential Oils from Three *Artemisia* Species against *Colletotrichum gloeosporioides* of Mango

**DOI:** 10.3390/antibiotics10111331

**Published:** 2021-11-01

**Authors:** Xing Huang, Tiantian Liu, Chunxiang Zhou, Yulin Huang, Xing Liu, Haibin Yuan

**Affiliations:** Department of Plant Protection, Jilin Agricultural University, Changchun 130118, China; huangxing@jlau.edu.cn (X.H.); ltt15944890963@163.com (T.L.); 18844145927@163.com (C.Z.); huangyulin0208@163.com (Y.H.); LX1065610622@163.com (X.L.)

**Keywords:** *Artemisia* species, *Colletotrichum gloeosporioides*, inhibitory effects

## Abstract

Post-harvest diseases of mango reduce fruit quality and cause severe yield losses with completely unmarketable fruits. The most common diseases of mangos are anthracnose (*Colletotrichum gloeosporioides*). In this study, the antibacterial activities of essential oils from *Artemisia scoparia*, *Artemisia lavandulaefolia*, and *Artemisia annua* against *C. gloeosporioides* were tested. The results showed that the essential oil of *A. scoparia* was more effective by the agar diffusion method; the EC_50_ value was 9.32 µL/mL. The inhibition rate was 100%, at a concentration of 10 μL/mL, through the spore germination method. The morphological changes of the mycelium were observed by scanning electron microscopy (SEM), the mycelia treated with essential oils showed shrinking, deformity, fracture, and dryness through SEM. *A*. *scoparia* essential oil was inoculated in vivo and subjected to paroxysm testing under natural conditions. *A. scoparia* had significantly inhibitory activity, and the inhibition rate was 66.23% in vivo inoculation tests after 10 days. The inhibition rate was 92.06% in the paroxysm test under natural conditions after 15 days. Finally, *A. acoparia* essential oil was analyzed by gas chromatography-mass spectrometry. The main compounds were 2-ethenyl-Naphthalene (23.5%), 2,4-pentadiynyl-Benzene (11.8%), 1,2-dimethoxy-4-(2-propenyl)-Benzene (10.0%), β-Pinene (8.0%), and 1-methyl-4-(1-methylethyl)-1,4-Cyclohexadiene (6.3%). The results have revealed the potential use of *A. scoparia* essential oil against post-harvest fungal pathogens *C. gloeosporioides*.

## 1. Introduction

Mango (*Mangifera indica* L.) is one of the most important tropical and subtropical fruit crops, with more than one hundred different cultivars [1]. Mango anthracnose is the most important fungal disease in the mango industry, and it is extremely harmful to mango fruit. Anthracnose is caused by *Colletotrichum gloeosporioides* (Penz.), and its life cycle begins when conidia attach to the fruit surface, germinate, and produce penetration structures [2]. This pathogen causes quiescent infections and remains latent until fruit and environmental factors favor the development of the disease and the onset of symptoms. Disease symptom may appear long after the initial stages of infection [3,4,5,6].

In mango production areas, anthrax can infect various plant organs, such as leaves, inflorescences, fruits (especially young fruits), and shoot tips. After the young leaves of the fruit trees are damaged, the formed lesions will bulge and finally perforate. It is difficult to control and may cause the fruit to rot during storage and transportation, shortening the shelf life of the fruit. The disease occurs as quiescent infections on immature fruit, and the damage is more important in the post-harvest period [7]. It is reported that anthracnose accounts for approximately 70% of mango post-harvest diseases [8,9]. Fungicides, either as pre- or post-harvest treatments, are the main approach to reduce losses from anthracnose [10]. However, the long-term use of chemical fungicides not only causes pathogens to become resistant to the fungicides, but it is also not conducive to food safety. Indiscriminate fungicide use may cause environmental pollution, including ecotoxicity to fish and health risks, because of its suspected carcinogenic properties [11,12]. Therefore, there is a need for environmentally friendly alternatives to managing this disease.

The use of biological agents is one of the strategies of interest [13]. To avoid chemical hazards, the use of natural compounds, produced by some plants, to control plant diseases is recommended [14,15]. Many research approaches indicate that essential oil and its constituents are effective antifungal agents. Essential oils are obtained from plant roots, stems, leaves, branches, fruits, seeds, flowers, and even whole plants. Essential oil is an oily, volatile substance and emits an obvious odor [16,17]. According to our research materials, the *Artemisia* species are one of the largest and most widely distributed genera of Asteraceae and consists of more than 350 species, of which, approximately 185 species are found in China [18,19]. Many species have medicinal value [20,21]. Its antifungal effect is also expressed in previous reports. Soylu et al. [22] found that *A. annua* exhibits an extremely strong activity against *Botrytis cinerea*, *Phytophthora infestans*, and *Verticilliumdahliae*, and, especially, against *Sclerotiniasclerotiorum*. In laboratory conditions, the inhibitory activity against *Fusarium. oxysporum *f. sp. *vasinfectum* and *Fusarium moniliforme* was conducted by Yan et al. [23]. As reported by Jiang et al. [24], the essential oil of *A. lavandulaefolia* had been proven to inhibit the mycelial growth of *Pyricularia grisea* and *Rhizoctonia solani*. This essential oil had also exhibited considerable antimicrobial activity against obligate anaerobic bacteria, according to Cha et al. [25]. Similarly, the essential oil of *A. scoparia* exhibited considerable inhibitory effects against all oral bacteria tested [26]. Additionally, Farzaneh et al. [27] tested the antifungal activity of *A. scoparia* essential oil against some soil-borne pathogenic fungi. Moreover, there were also reports on the antifungal effect of essential oils obtained from the genus *Artemisia* [28,29,30,31]. Particularly, essential oil can serve as an alternative means of control and may be used as a botanic fungicide against post-harvest fungal pathogens in the future [32,33]. Essential oil obtained from black caraway, fennel, peppermint, garlic, and wood ash expressed as highly effective against sweet cherry fruit [34] and four post-harvest rots of banana [35]. The objectives of this study were to test and compare the inhibitory effects of the essential oils from *A. scoparia*, *A. lavandulaefolia,* and *A. annua* against the causative agent of *C. gloeosporioides*, as well as to evaluate potential applications of essential oils for controlling the post-harvest disease for mango fruit.

## 2. Results

### 2.1. The Antifungal Activities by the Agar Diffusion Method

Among all essential oils tested, *A. scoparia* essential oil caused the greatest inhibition of the mycelium growth of *C. gloeosporioides*. The EC_50_ (the concentration inhibiting mycelium growth by 50%) values of each essential oil were also estimated using probit analyses. The lowest EC_50_ values of the contact phases of the essential oils were recorded for *A. scoparia* (9.320 μL/mL), followed by *A. lavandulaefolia* (19.064 μL/mL) and *A. annua* (30.278 μL/mL) (Table 1).

### 2.2. Evaluation of the Antifungal Activity of Plant Essential Oils Delivered In Vitro Fumigation 

With different concentrations of essential oil of the *Artemisia* species, the percentage of the inhibition of mycelial growth was remarkable for all tested. This result suggests that essential oils have significant activity (*p* < 0.05) and inhibit the mycelial growth. The lowest EC_50_ values of essential oils were for *A. scoparia* (6.464 μL/plate), followed by *A. lavandulaefolia* (9.485 μL/plate) and *A. annua* (16.194 μL/plate) (Table 2).

### 2.3. Effect of Essential Oils on Conidial Germination

**Figure 1:**
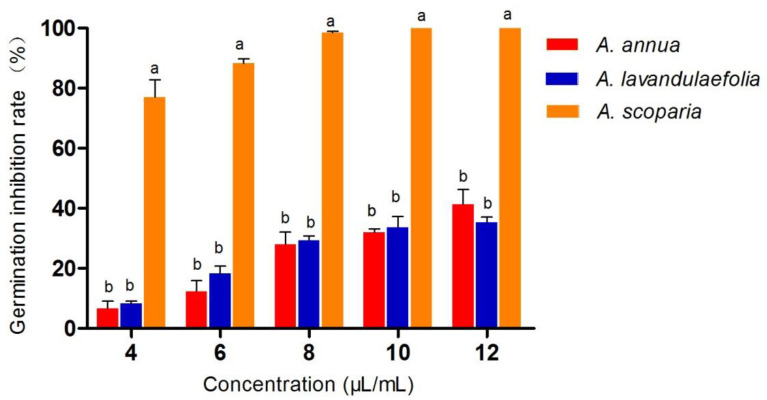
Effects of essential oils spore germination of *C. gloeosprioides* values, followed by the same letters (a, b) are not significantly different at 5%.

The effects of different concentrations of three *Artemisia* species’ essential oils on the conidial germination of *C. gloeosporioides* were assessed (Figure 1). *A. scoparia* essential oil was found to be most active on the germination of spores. Complete inhibition of conidial germination by *A. scoparia* essential oil was observed at a 10 μL/mL concentration, whereas *A. lavandulaefolia* and *A. annua* essential oil could inhibit this activity by 30% and 40%.

### 2.4. Determination of the Effect of Essential Oils on Hyphal Morphology

**Figure 2:**
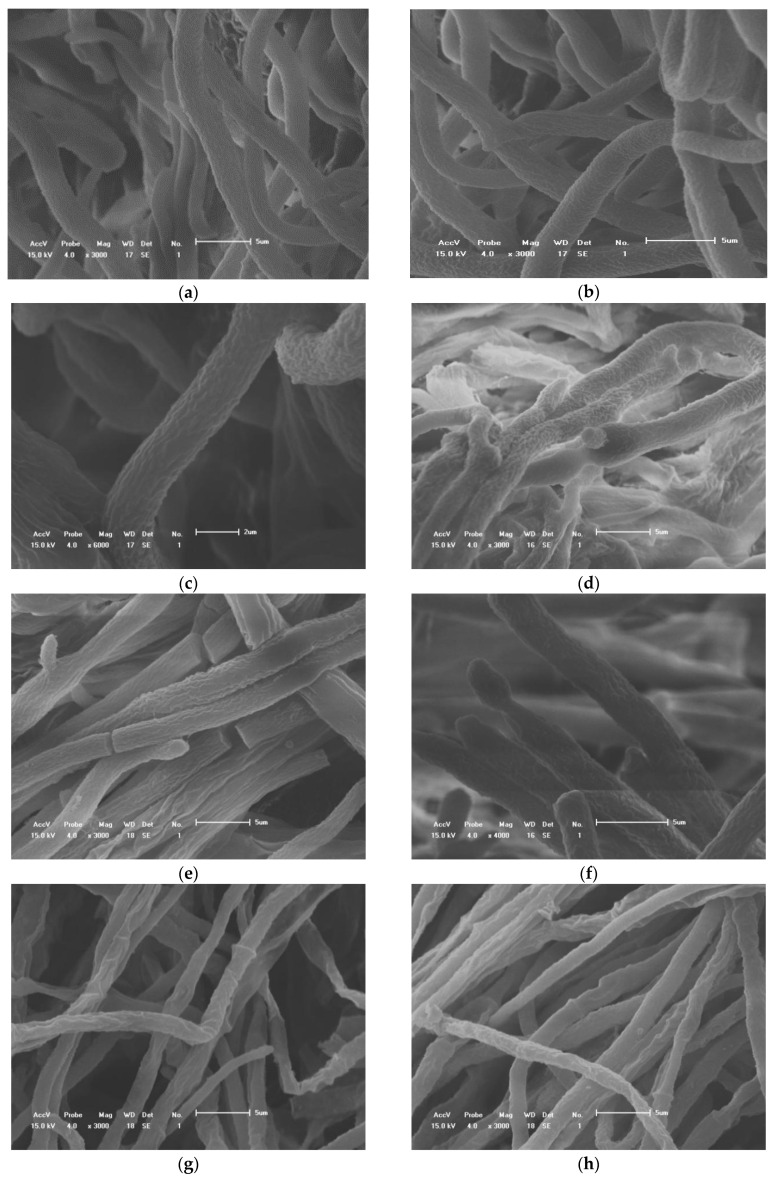

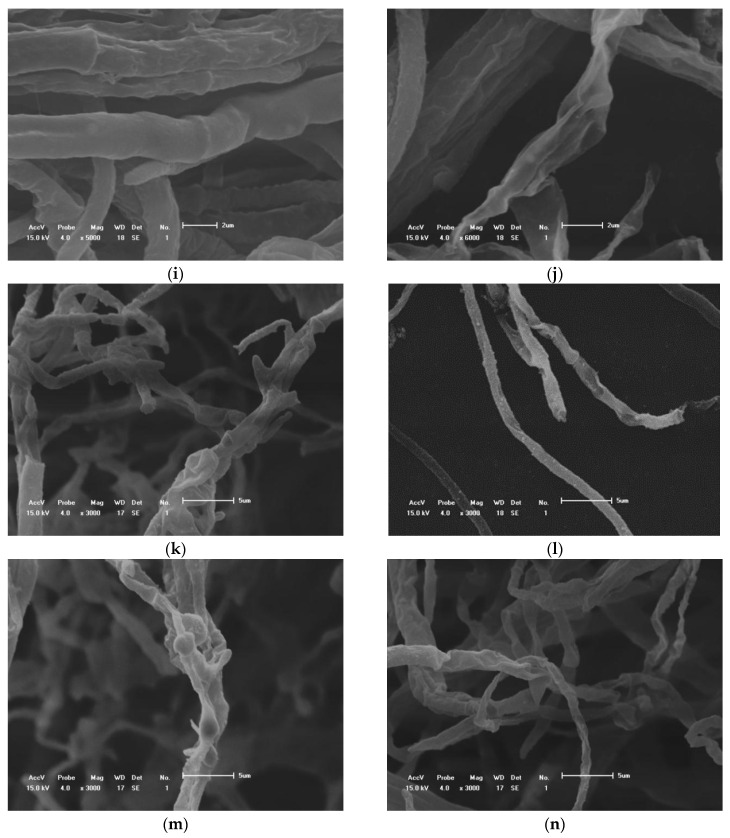
Scanning electron microscopy of the hyphae exposed to three essential oils; (**a**–**c**): controls; (**d**–**f**): *A. annua*; (**g**–**i**): *A. lavandulaefolia*; (**j**–**n**): *A. scoparia*.

The hyphae of all treatment groups were treated with 15 μL/mL. Observed by scanning electron microscope, the hyphae of the control group were full and uniform in thickness. After treatment with *A. annua* essential oil, the thickness of the hyphae varied with overflow shrinkage, and the top of the hyphae was deformed and locally enlarged. After the treatment of the essential oil from *A. lavandulaefolia*, the thickness of the hyphae was uneven, the hyphae were sunken, severely shrunk, and shriveled, the hyphae were broken, and the contents leaked out. After the treatment with *A. scoparia* essential oil, the mycelium branched abnormally, locally overflowed, and expanded, the hyphae broke, the contents leaked out to form empty tube hyphae, and the hyphae appeared severely twisted and clumped (Figure 2).

### 2.5. In Vivo Tests of the Volatile Compounds Produced by A. scoparia Essential Oil

The development and expansion of disease symptoms induced by the anthracnose pathogen were inhibited effectively by *A. scoparia* essential oil in vivo screens. For the inoculated control fruit, the lesion area extended to 12.54 cm^2^ after 10 days of incubation at room temperature; whereas, for the inoculated fruits exposed to volatiles from 50 μL and 120 μL of *A. scoparia*, essential oil areas were limited to 8.55 and 4.93 cm^2^, and the control value reached at 31.81% and 66.23% (Table 3).

### 2.6. The Effect of A. scoparia Essential Oil on Natural Morbidity

The application of 120 μL *A. scoparia* essential oil on mangos caused a decay inhibition of 92.06%, and the disease index was 4.44%. The control group disease index was 82.22%, and a difference was observed for the fruits (Table 4).

### 2.7. The Components of A. scoparia Essential Oil 

**Figure 3:**
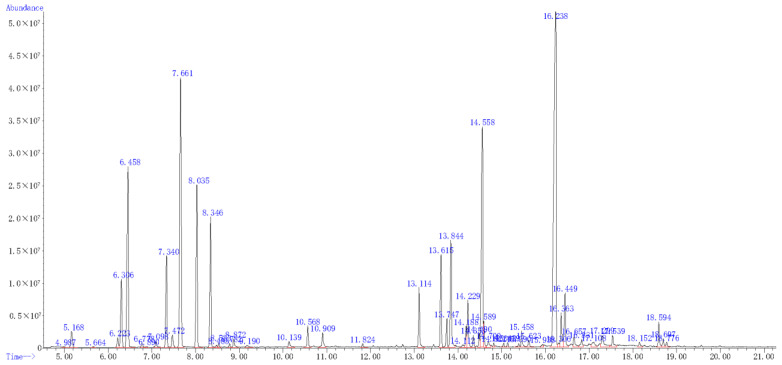
Total iron current chromatogram of the essential oil from *A. scoparia*.

The essential oil yield of *A. scoparia* was 0.64% (*v*/*w*) and a total of 45 components were identified, accounting for 95.4% of the total oil. The main compounds were 2-ethenyl-Naphthalene (23.5%), 2,4-pentadiynyl-Benzene (11.8%), 1,2-dimethoxy-4-(2-propenyl)-Benzene (10.0%), β-Pinene (8.0%), and 1-methyl-4-(1-methylethyl)-1,4-Cyclohexadiene (6.3%) (Figure 3 and Table 5).

## 3. Discussion

The potential use of essential oils for developing promising fungicidal agents has been focused on by many researchers [36]. The essential oil of *Pinus pinea* had previously demonstrated significant fungicidal activity, inhibiting the growth of ten plant pathogenic fungi. Among them, the imbibition rate was 63.7% against *Fusarium oxysporum* at 4 μL/mL [37]. Bocate et al. [38] also found that *Fusarium verticillioides* was totally inhibited by ≥5 μL/L of garlic essential oil, and 2.5 μL/L was capable of inhibiting 72% of the growth in vitro fumigation. According to Combrinck et al. [39], the antifungal properties of eighteen essential oils were evaluated in vitro, and the results showed that thyme oil was most effective against *Lasiodiplodia theobromae*, isolated from mango, and caused total inhibition of the pathogen at a concentration of 200 μL/L. Bista et al. [40] reported the essential oil of *Cinnamomum tamala* showed the best performance of anti-fungal effect in controlling *C. gloeosporioides*, which inhibited the mycelial growth by 95.45% at 40 μL/mL concentrations. In our study, the essential oil of *A. scoparia* was more effective, and the EC_50_ value was 9.32 µL/mL by the agar diffusion method. Plant essential oils not only inhibit the growth of hyphae but also inhibit spore germination. Our findings revealed that *A. scoparia* essential oil expressed complete inhibition of conidial germination at 10 μL/mL concentrations. Consistently, essential oils obtained from the *Lippia sidoides*, *Ocimum gratissimum, Lippia citriodora*, and *Cymbopogon citratus* plants had an effect on *C. gloeosporioides* conidia germination, as reported by Júnior et al. [41].

Plant essential oil inhibits the growth of mycelium and causes changes in mycelial morphology. Similar observations were recently presented by Tripathi et al. [42], who found that *Hyptis suaveolens* essential oil caused severe damage and alterations to the vegetative hyphae of *Fusarium oxysporum* f. sp. The observations, made with light and electron microscopy, were in accordance with previous studies, in which essential oils of aromatic plants caused morphological alterations in the fungal hyphae [43,44,45]. In our research, SEM observations of *C. gloeosporioides* hyphae, exposed to essential oils, revealed alterations in the hyphal morphology. Shriveled hyphal aggregates were commonly observed in essential oil-treated mycelium, compared with the thick, elongated, and normal mycelial growth of the controls.

The application of essential oils in post-harvest preservation is currently in the spotlight, due to their organic, safe, and effective controlling nature. Sefu et al. [46] and Palhano et al. [47] found that cinnamon and ginger essential oil showed high antifungal effects on mycelia growth of mango anthracnose disease-causing fungi. Chen et al. [48] conducted the antifungal assay of the essential oil against *Alternaria alternata* in vivo on cherry tomato, the disease incidence at oil concentrations of 0.2–1.5 μL/mL was 88–48%. In our study, *A. scoparia* had significant inhibitory activity and the inhibition rate was 66.23% in vivo inoculation tests after 10 days. In naturally infected fruit, there was a significant reduction in the incidence of decay with the application of thyme essential oils. The highest concentration (0.15%) completely controlled *C. gloeosporioides*, according to Bosquez-Molina et al. [49]. Shehata et al. [50] revealed that essential oil treatments with orange, lemon, and mandarin extended the shelf-life of strawberries and delayed their deterioration for up to 18 days. Our study found that the essential oil of *A. scoparia* also showed a strong effect on *C. gloeosporioides*, and the inhibition rate was 92.06% in the paroxysm test under natural conditions after 15 days.

Essential oils can be used as alternatives for the currently-used fungicides because they are rich in biologically active chemicals [51,52]. In our study, β-Pinene is one of the main components in *A. scoparia* essential oil and accounts for 8% of the total ingredients. The chemical compositions of the essential oils reported here were in partial agreement with previous reports, as Negahban et al. [53] reported that the main constituents of the oil from plants during the blooming period were β-pinene (19.01%), capillin (17.45%), limonene (15.11%), and myrcene (10.95%) in the Tehran Province. Another study documented that 1-phenyl-penta-2,4-diyne (30.9%), β-pinene (23.3%), and limonene (10.2%) were the predominant constituents of the oils in central Iran [54]. Due to the extraction method and geographic and seasonal factors, there are some variations in the chemical compositions among them [55,56]. β-myrcene (30.2%), β-cymene (12.8%), and (+)-limonene (12.4%) were the main monoterpenes of the fresh leaves of *A. scoparia*, collected during the last week of June in northern India (Chandigarh) [57]. Moreover, the major components from the volatile oils were β-myrcene (24.4%), β-terpinene (18.3%), and neral (12.5%) in northern India (New Delhi) [58]. Essential oils have very complex ingredients, containing 20–60 components in different concentrations [59,60]; previous studies have been conducted on which ingredients work. Liu et al. [61] demonstrated *Artemisia absinthium* L. and its isolation from supercritical fluid extraction, thiophenes, played an important role in antifungal activities. Another study revealed that *Artemisia absinthium* L. essential oil showed significant inhibitory activity against *Sclerotinia* (23.61%) and *Rhizoctonia solani* (25.39%). Additionally, its major component, chamazulene, was attributed to the antifungal activity [62]. Morover, Montenegro et al. [32] evaluated the antifungal activities of *Mentha pulegium* essential oil and its major constituents. *M. pulegium* essential oil and isopulegol exhibited the highest antifungal activity against *Monilinia fructicola* and *Botrytis cinerea*. However, the antifungal activities of the main constituents of the essential oils were not examined in our investigation. Further studies are required to characterize those components of the essential oils for additional screening, so that their potential applications in controlling plant disease can be fully exploited.

## 4. Materials and Methods

### 4.1. Plant Material and Essential Oil Extraction

Fresh aboveground parts of *A. lavandulaefolia*, *A. scoparia*, and *A. annua* were collected in September 2019 from plants at the flowering stage in Changchun (43.8170°N, 125.3235°E). The identity was confirmed by Dr. Tong-Bao Qu, College of Horticulture, Jilin Agricultural University. Voucher specimens (*A. lavandulaefolia*: JLH 2158; *A. scoparia*: JLH 1681; *A. annua*: JLH 2251) were deposited at the Herbarium of the College of Horticulture, Jilin Agricultural University. Each plant was crushed and then dried separately in the shade at ambient temperature. Each 1.5 kg was soaked in water for 12 h with a solid: liquid ratio of 1:1, after which the aerial parts were subjected to hydrodistillation for 3 h using a clevenger-type apparatus; the residue was also repeated three times. Finally, the oil was dried over anhydrous sodium sulfate and stored in a sealed vial at 4 °C.

### 4.2. Preparation of Colletotrichum Gloeosporioides

The plant pathogenic fungi, *C. gloeosporioides*, was provided by the Department of Plant Pathology, College of Plant Protection, Jilin Agriculture University, these were maintained on potato dextrose agar (PDA) medium for 5–7 days at 28 °C until uniform mycelial growth was obtained.

### 4.3. Investigation of Antifungal Activities by the Agar Diffusion Method

For the determination of contact effects, the essential oils were dispersed as an emulsion in water using Tween-80 (0.05%) and added to PDA, immediately before they were emptied into glass petri dishes (90 mm in diameter) at a temperature of 40–45 °C. The concentrations tested were 4, 6, 8, 10, and 12 μL/mL. The 1 mL 0.05% Tween-80 solution was mixed with PDA, as controls. *C. gloeosporioides* was inoculated immediately by plating to the center of each plate with an 8 mm diameter disc of the fungus and cut with a sterile cork borer from the edge of actively growing cultures on PDA plates. The mycelium growth was measured. The colony diameter of each plate was measured by the cross method, all the treatments were compared with the control. Thirty plates were tested for each treatment and repeated thrice. The EC_50_ was obtained for each treatment by fitting % Inhibition and concentration to a dose–response equation. The inhibition rate of treatments against the control was calculated by percentage, according to the following formula:

Growth inhibition (%) = (C − T)/(T − 8) × 100(1)

where C is the radial growth of the tested fungus in the control (mm), and T is the radial growth of the tested fungus in the treatment (mm).

### 4.4. Evaluation of the Antifungal Activity of Plant Essential Oils Delivered In Vitro Fumigation

To determine the inhibitory effects of the essential oils, molten PDA medium was poured into petri plates (90 mm in diameter) containing 9 mL of warm sterilized PDA medium. A PDA plug, with a diameter of 8 mm, taken from actively growing cultures, was placed in the center of the culture medium, and sterilized filter papers (6 mm in diameter) with different amounts of plant essential oils (4, 6, 8, 10, and 12 μL/plate) were separately placed on the lid. Filter paper without essential oil was prepared as a control. All the treatments were compared with control. Thirty plates were tested for each treatment and repeated thrice. Each plate was sealed with parafilm to prevent the leakage of essential oils. All experiments were incubated at 28 ± 1 °C for 5–7 days. The colony diameter was measured by the cross method after the colony diameter of the blank control was more than two-thirds the diameter of the plate. The EC_50_ was obtained for each treatment by fitting % inhibition and concentration to a dose–response equation. The inhibition rate of treatments against the control was calculated by percentage, according to the Equation (1).

### 4.5. Spore Germination Assays

The effects of essential oils on spore germination were assessed. A spore suspension (10^6^ spores/mL) of *C. gloeosporioides* was prepared from an actively growing culture (7–8 days old) in distilled sterile water. Three different 20 μL aliquots of the spore suspension drops were spread onto the surface of the PDA medium supplemented with different concentrations of essential oils in contact phases, as described before. Sterile distilled water, containing 0.05% Tween-80, was used in place of the essential oils as controls. Thirty plates were tested for each treatment and repeated thrice. Each plate was sealed with parafilm to prevent the leakage of essential oils. Plates were incubated at 28 °C until the germination in the control reached >85% (10–12 h, according to the rate of germination of the fungus). Afterward, spore germination was stopped by applying a drop of lactophenol cotton blue to the inoculation sites on the plates. Germination was defined as the point at which the germ tube length exceeded the spore diameter. The percentage of spore germination and the lengths of the germ tubes, three replicates were conducted for each treatment, each replicate was estimated under a microscope at a minimum of 300 spores using a micrometer.

Conidial germination inhibition (%) = [(Gc − Gt)/Gc)] × 100

where Gc and Gt represent the mean number of germinated conidia in the control and treated petri plates.

### 4.6. The Determination of the Effect of Essential Oils on Hyphal Morphology

For SEM analysis, fungal mycelia were processed as follows: mycelial discs (1 cm in diameter) were fixed with 2.5% glutaraldehyde in 0.1 M phosphate buffer (pH 7.0) for 4 h at room temperature. They were washed three times for 10 min, each time in the same buffer. After fixation, the samples were dehydrated in a graded ethanol series (30, 50, 70, 80, 90, 95, and 100%, three times) for 15 min in each series. The samples were freeze-dried. Fixed material was then mounted on stubs using double-sided carbon tape and coated with gold/palladium in a sputter coater system in a high-vacuum chamber (Polaron SC7620, Quorum Technologies, Laughton, East Sussex, UK) for 150 s at 9 mA. The samples were examined, and digital images were captured using a JEOL JSM 5500 SEM at an accelerating voltage of 5 kV.

### 4.7. In Vivo Assay

Freshly harvested, mature, and healthy mangos, with approximately equal size and color, were used in this experiment. The fruits were free from visible defects and rot. Before each trial, the mangos were washed with water and sterilized with 2% sodium hypochlorite for 10 s, then rinsed under running sterile water for 1 min and air-dried. Each fruit was wounded with a 6 mm diameter hole-puncher and injected with 6 mm diameter mycelial plugs of *C. gloeosporioides* in the wound area. To study the antagonistic activity of volatiles on mycelia growth, ten inoculated fruits were arranged in a container (30.5 cm × 22 cm × 14 cm) as one replicate. Additionally, 50, 80, 100, and 120 μL of *A. scoparia* essential oil were placed in the container, without direct contact with the mangos. Thirty mangos were tested for each treatment and repeated thrice; distilled water was taken to the container as controls. After 10 days at 28 °C, the diameters of the decayed spots were measured, and the lesion area was calculated to contrast the treatment effects. The inhibition rate of the treatments against the control was calculated by percentage, according to the following formulas:

Rot area (mm^2^) = π × (rotted symptom radius)^2^

Control value(%) = (rot area of untreated fruit-rot area of treated fruit)/rot area × 100

### 4.8. Effect of Essential Oil Fumigation on Decay Incidence and Mango Fruit Quality

Ten fruits were arranged in a container (30.5 cm × 22 cm × 14 cm) as one replicate. Additionally, 50, 100, and 120 μL of *A. scoparia* essential oils were placed in the container, without direct contact with the mangos. Distilled water was added to the container as control groups. Thirty mangos were tested for each treatment and repeated thrice. After 15 days at 28 °C, the diameters of the decayed spots were measured, and the lesion area was calculated to contrast treatment effects. The inhibition rates of the treatments against the control were calculated by percentage.

Disease index (%) = 100 × ∑[(Number of melons affected × Incidence level)/(Total number of melons × Highest level)]

The disease severity index of anthracnose on the mangos was rated on a scale of 0–9 (0 = no disease symptom, 1 = 0.1–5%, 3 = 5.1–15%, 5 = 16–25%, 7 = 26–50%, and 9 = 51–100%), as the percentage of diseased mango area.

### 4.9. Determination of Chemical Composition of Essential Oil from A. scoparia

The constituents of the *A. scoparia* essential oil was confirmed by gas chromatography using a GC system (Agilent 6890N, Agilent Technologies Incorporated, California, United States), which was equipped with an HP-1 capillary column (30 m × 0.25 mm × 0.25 μm film thickness). The oven temperature was programmed at 60 °C for 3 min, with an increase of 10 °C/min, until 280 °C for 5 min. The carrier gas was helium at a flow rate of 1.0 mL/min, the split ratio was 50:1, and the injection volume was 1.0 μL.

The mass spectrometer (Agilent 5975N, Agilent Technologies Incorporated, Palo Alto, CA, USA) used an electron ionization source with 70 eV ionization energy. The ion source temperature was 230 °C, with a scanning range between 20 and 650 *m*/*z*. The temperature of the quadrupole was 150 °C, and the mass spectrum acquisition delay time was 2 min. The constituents were identified based on their retention index and the use of the mass spectral libraries (National Institute of Standards and Technology, NIST databases). The area normalization method was used to calculate the relative content of each constituent.

### 4.10. Statistical Analysis

All the experiments were performed in triplicate and the calculated data were expressed as mean ± standard deviation. Statistical analysis of the data were completed by using SPSS Statistics 17.0 (IBM, New York, NY, USA) software. The differences between the components were evaluated using ANOVA, Tukey’s HSD test at 5% significance.

## 5. Conclusions

Our study indicated that *A. lavandulaefolia, A. scoparia*, and *A. annua* essential oils possessed antifungal activity against *C. gloeosporioides* in mangos. Furthermore, the antibacterial mechanism was preliminarily investigated. The ultra-depth microscope and SEM showed that the mycelium of *C. gloeosporioides* treated with *Artemisia* species essential oil was severely deformed, the mycelium was broken, and the contents were leaked to form an empty tube. *A. scoparia* essentail oil was inoculated in vivo and subjected to paroxysm testing under natural conditions, it had significantly inhibitory activity; the inhibition rate was 66.23% in vivo inoculation tests after 10 days. The inhibition rate was 92.06% in the paroxysm tests under natural conditions after 15 days. This study determined the effectiveness of essential oils from *A. scoparia* against the decay of mangos to extend the shelf life of mango fruits post-harvest.

## Figures and Tables

**Table 1:** EC_50_ value of three essential oils against *C. gloeosprioides*, by the agardiffusion method (μL/mL).

Essential Oil	Virulence Regression Equation	Correlation Coefficient (r)	EC_50_
*A. annua*	y = −2.051 + 0.068x	0.929	30.278
*A. lavandulaefolia*	y = −1.600 + 0.84x	0.994	19.064
*A. scoparia*	y = −2.106 + 0.226x	0.954	9.320

**Table 2:** EC_50_ value of three essential oils from *Artemisia* species against *C. gloeosprioides* in vitro fumigation (μL/plate).

Essential Oils	Virulence Regression Equation	Correlation Coefficient (r)	EC_50_
*A. annua*	y = −1.161 + 0.72x	0.978	16.194
*A. lavandulaefolia*	y = −0.689 + 0.73x	0.886	9.485
*A. scoparia*	y = −0.521 + 0.81x	0.755	6.464

**Table 3:** Inhibitory effects of *A.scoparia* essential oil against *C. gloeosporioides* in vivo tests.

Concentrations (μL)	Rot Area (cm^2^) ± SD	Control Value (%)
CK	12.54 ± 0.56 ^a^	-
50.00	8.55 ± 0.33 ^b^	31.81
80.00	3.99 ± 0.48 ^d^	38.07
100.00	4.77 ± 0.16 ^bc^	65.66
120.00	4.93 ± 0.57 ^cd^	66.23

All data were expressed as the mean ± standard deviation. The values with different superscripts ^(a–d)^ in the same column are significantly different (ANOVA, Tukey’s HSD test at 5% significance).

**Table 4:** Inhibitory effects of *A.scoparia* essential oil against *C. gloeosporioides* on natural morbidity.

Concentrations (μL)	Rot Area (cm^2^) ± SD	Disease Index (%)	Control Value (%)
CK	47.09 ± 7.10 ^a^	82.22	-
50.00	8.51 ± 3.72 ^b^	22.22	82.39
100.00	7.31 ± 2.70 ^b^	17.77	85.18
120.00	3.84 ± 3.07 ^b^	4.44	92.06

All data were expressed as the mean ± standard deviation. The values with different superscripts ^(a, b)^ in the same column are significantly different (ANOVA, Tukey’s HSD test at 5% significance).

**Table 5:** Chemical composition of essential oil from *A. scoparia*.

No.	Compounds	RI	Percent Composition (%)
1	1R-πPinene	948	0.7
2	β-Pinene	943	8.0
3	3,3,6-Trimethyl-1,4-heptadien-6-ol	983	0.2
4	Limonene	1018	3.3
5	(E)-3,7-dimethyl-1,3,6-Octatriene	976	0.6
6	2-methyl-5-(1-methylethenyl)-2-Cyclohexen-1-ol	1206	2.0
7	1-methyl-4-(1-methylethyl)-1,4-Cyclohexadiene	998	6.3
8	3,3,6-trimethyl-1,5-Heptadien-4-one	1042	4.6
9	3,3,6-Trimethyl-1,5-heptadien-4-ol	1068	0.1
10	1-methyl-4-(1-methylethylidene)-Cyclohexene	1052	0.2
11	2-ethenyl-1,1-dimethyl-3-methylene-Cyclohexane	1071	0.1
12	3,7-dimethyl-1,6-Octadien-3-ol	1082	0.4
13	2,6-dimethyl-3,7-Octadiene-2,6-diol	1197	0.1
14	(R)-5-methyl-2-(1-methylethenyl)-4-Hexen-1-ol	1146	0.3
15	(R)-4-methyl-1-(1-methylethyl)-3-Cyclohexen-1-ol	1137	0.8
16	ππ-trimethyl-3-Cyclohexene-1-methanol	1143	0.7
17	Acetate 5-methyl-2-(1-methylethenyl)-4-Hexen-1-ol	1270	0.1
18	2,4-pentadiynyl-Benzene	1206	11.8
19	Caryophyllene	1424	3.2
20	(Z)-7,11-dimethyl-3-methylene-1,6,10-Dodecatriene	1440	0.9
21	Eugenol	1392	4.0
22	πCaryophyllene	1456	0.7
23	(R)-2,4a,5,6,7,8-hexahydro-3,5,5,9-tetramethyl-1*H*-Benzocycloheptene	1497	1.6
24	(Z,E)-3,7,11-trimethyl-1,3,6,10-Dodecatetraene	1486	0.4
25	[S-(R*,S*)]-5-(1,5-dimethyl-4-hexenyl)-2-methyl-1,3-Cyclohexadiene	1492	0.4
26	1,2-dimethoxy-4-(2-propenyl)-Benzene	1361	10.0
27	Octahydro-7-methyl-3-methylene-4-(1-methylethyl)-,3aS,3bR,4S,7R,7aR)-1*H*-Cyclopenta[1,3]cyclopropa[1,2]benzene	1339	0.6
28	[4aR-(4aπ7π8aπ]-decahydro-4a-methyl-1-methylene-7-(1-methylethenyl)-Naphthalene	1469	0.3
29	1-ethenyl-1-methyl-2-(1-methylethenyl)-4-(1-methylethylidene)-Cycloheane	1431	0.2
30	(1S-cis)-1,2,3,5,6,8a-hexahydro-4,7-dimethyl-1-(1-methylethyl)-Naphthalene	1469	0.2
31	[1R-(1π3aπ4π8aπ]-decahydro-1,5,5,8a-tetramethyl-1,4-Methanoazulen-9-one	1576	0.2
32	2-methylene-6,8,8-trimethyl-Tricyclo[5.2.2.0(1,6)]undecan-3-ol	1233	0.2
33	(E)-3,7,11-trimethyl-1,6,10-Dodecatrien-3-ol	1564	0.6
34	Octahydro-3,6,6,7a-tetramethyl-2*H*-2a,7-Methanoazuleno[5,6-b]oxirene	1293	0.3
35	o-Hydroxybiphenyl	1456	0.7
36	2-ethenyl-Naphthalene	1367	23.5
37	(−)-Spathulenol	1536	1.3
38	Caryophyllene oxide	1507	2.0
39	5-Hydroxy-4,4-dimethyl-1,5-diphenylpent-1-yn-3-one	2294	0.6
40	1,5,5,8-tetramethyl-[1R-(1R,3E,7E,11R)]-12-Oxabicyclo[9.1.0]dodeca-3,7-diene	1592	0.4
41	Cubenol	1580	0.5
42	[2R-(2π4aπ8aπ]-decahydro-ππ4a-trimethyl-8-methylene-2-Naphthalenemethanol	1593	1.0
43	Phenol, 2-methoxy-4-(2-propenyl)-, acetate	1552	0.6
44	1-phenyl-2,4-Hexadiyn-1-one	1461	0.3
45	8a-dimethyl-6-(1-methylethenyl)-2(1*H*)Naphthalenone,3,5,6,7,8,8a-hexahydro-4	1673	0.3

## Data Availability

Data is contained within the article.
